# T cell fate mapping and lineage tracing technologies probing clonal aspects underlying the generation of CD8 T cell subsets

**DOI:** 10.1111/sji.12983

**Published:** 2020-10-26

**Authors:** Shaima Al Khabouri, Carmen Gerlach

**Affiliations:** ^1^ Division of Rheumatology Department of Medicine Karolinska Institutet Karolinska University Hospital Stockholm Sweden; ^2^ Center for Molecular Medicine Karolinska University Hospital Solna Stockholm Sweden

**Keywords:** fate mapping, lineage tracing, single‐cell technologies, T cell subsets

## Abstract

T cells responding to acute infections generally provide two key functions to protect the host: (1) active contribution to pathogen elimination and (2) providing long‐lived cells that are poised to rapidly respond to renewed infection, thus ensuring long‐lasting protection against the particular pathogen. Extensive work has established an astonishing amount of additional diversity among T cells actively contributing to pathogen elimination, as well as among resting, long‐lived antigen‐experienced T cells. This led to the description of a variety of functionally distinct T cell ‘subsets’. Understanding how this heterogeneity develops among T cells responding to the same antigen is currently an active area of research, since knowledge of such mechanisms may have implications for the development of vaccines and immunotherapy. The number of naïve T cells specific to a given antigen span a great range. Considering this, one mechanistic angle focusses on how individual naïve T cells contribute to the development of the distinct T cell subsets. In this review, we highlight the current technologies that enable one to address the contributions of individual naïve T cells to different T cell subsets, with a focus on CD8 T cell subsets generated in the context of acute infections. Moreover, we discuss the requirements of new technologies to further our understanding of the mechanisms that help generate long‐lasting immunity.

## 1 INTRODUCTION

**FIGURE 1:**
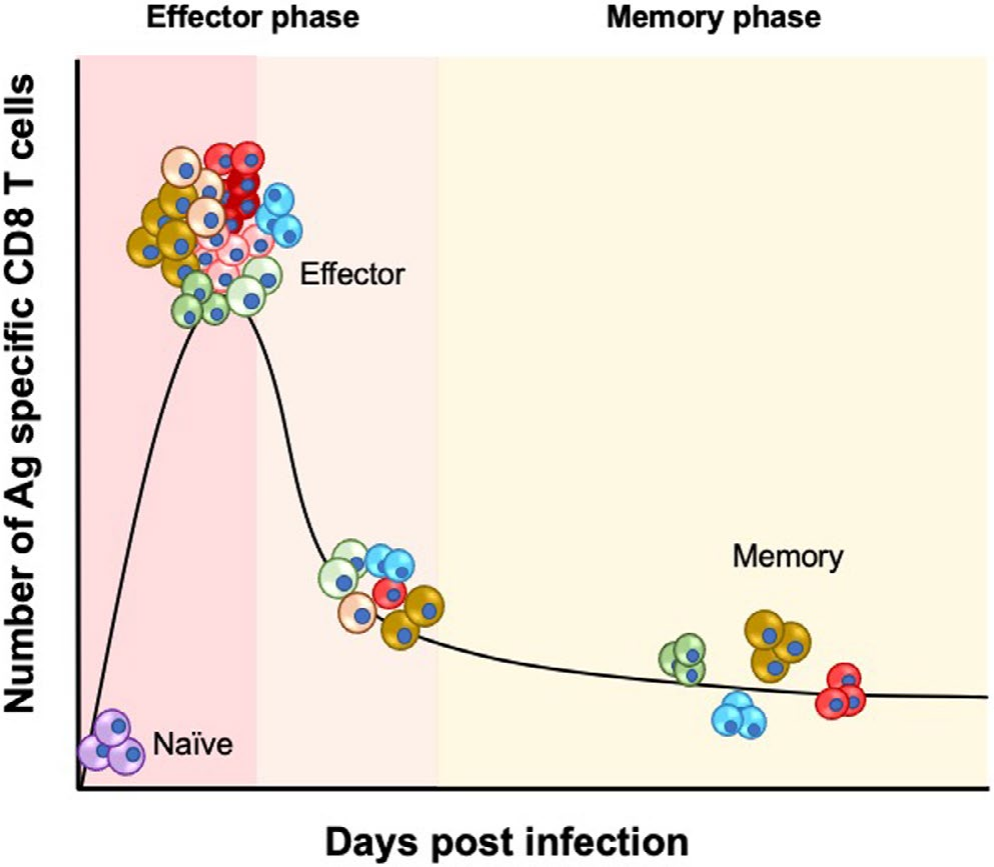
The different phases of a CD8 T cell response after an acute infection. A schematic representing the development of the CD8 T cell response and various effector and memory subsets following an acute infection. Naïve CD8 T cells recognize their antigen and undergo clonal expansion and proliferate, rapidly increasing in number. During this expansion, the T cells acquire different effector functions to eradicate the infection effectively. At this stage, the population of effector cells is heterogenous and different cells have different functional and phenotypic properties. Once the infection has been cleared, the response enters a contraction phase where most of the effector cells generated at the effector stage die. The expansion and contraction phases constitute the effector phase. The cells that are left behind are long‐lived and enter the memory phase. The population in the memory phase is also heterogeneous and comprised of populations of cells with different functional properties poised to act upon re‐infection

The primary T cell response to an acute infection follows a consistent pattern, which is summarized in Figure 1. Firstly, a naïve T cell population recognizes an antigen, and the cells undergo clonal expansion and bolster the original T cell population to manage the infection more effectively. During this expansion, the T cells differentiate and acquire effector functions which enable them to eradicate the pathogen quickly and efficiently during an acute infectious assault. Once the pathogen has been cleared, the T cell population contracts and most of the expanded T cells die, leaving only a relatively few long‐lived T cells. The periods of expansion and contraction constitute what we refer to in this review as the ‘effector phase’ and the cells found during this phase will be referred to as ‘effector T cells’. The long‐lived cells that remain after the effector phase we refer to as ‘memory T cells’. Neither the effector nor the memory T cell populations generated after an acute infection are homogeneous, but in fact are composed of several subsets with different functional properties. Seminal studies by Hamann et al^1^ and Sallustso et al^2^ identified subsets of antigen‐experienced CD8 T cells that differ phenotypically and functionally. Further studies have shown that these subsets have different migratory patterns,^3^, ^4^ suggesting that different subsets of effector and memory T cells play different and possibly unique roles in managing infections, and in providing long‐term protection. The generation of effector and memory T cell subsets has therefore garnered great interest among immunologists, propelling the identification and characterization of additional subsets and investigating the mechanisms by which these various subsets develop. Additionally, the number of naïve T cells that are primed to take part in the T cell response to a given antigen is tens to thousands of naive T cells per mouse^5^, ^6^ and 20 000 to 200 000 per person.^7^ These observations have prompted questions on whether individual T cells play unique roles in shaping the overall T cell response, and whether individual naive T cells are predisposed to generate a specific effector or memory T cell type. Indeed, several studies have demonstrated how individual naive T cells with the same antigen specificity are not in fact predisposed to give rise to a particular T cell subset, but rather are capable of generating a diverse range of effector and memory T cells.^8^, ^9^, ^10^, ^11^, ^12^ Furthermore, the responses of individual naïve T cells—even if they bear the same T cell receptor (TCR)—are not identical, but somewhat skewed towards the generation of certain phenotypes and longevity properties.^13^, ^14^, ^15^, ^16^ However, the T cell response is remarkably reproducible on a population level despite the different responses of each naïve T cell; the overall number of cells generated during a response, the size of the effector and memory populations and the functions gained during an acute infection are consistent per infection, and this reproducibility on a population level is attributed to an averaging of the different individual naïve T cell responses.^13^, ^14^, ^15^ Moreover, individual T cells themselves were found to control and regulate the size and phenotype of the responding T cell population through quorum sensing mechanisms.^17^, ^18^ Together, these observations highlight the complex regulation that T cells undergo—on both an individual and population level—to ensure an effective response to acute infection, as well as the generation of a robust memory response to tackle any future encounters.

Given the high variability in responses that individual T cells mount, understanding the regulation T cells undergo during a response requires studying individual naïve T cells spatially and temporally to monitor how the individual T cells participating in a response contribute to the diverse effector and memory populations generated. This also requires following the progeny of these individual T cells to investigate their role in shaping the overall T cell population and response to infection. In this review, we discuss the current technologies that allow for individual T cell monitoring and lineage tracing, and how these technologies have contributed to our current knowledge of T cell subset generation. In addition, we discuss how these technologies can be combined and/or improved to further our understanding of the processes required to generate the various effector and memory T cell subsets required for long‐lasting immunity.

## 2 CURRENT EFFECTOR AND MEMORY CD8 T CELL SUBSETS

Effector and memory CD8 T cells, as well as their subsets are traditionally classified based on their unique functional properties and migratory characteristics. However, determining cell migration patterns is difficult, and establishing true functional abilities in vivo is challenging. To overcome these obstacles, a variety of criteria have been used as surrogates of true functional and migratory properties to delineate the various CD8 T cell subsets; Phenotypic markers, transcriptomic and epigenetic profiles, in vivo cell behaviour, and in vitro stimulation and migration assays have been used to describe several T cell subsets.

### 2.1 Memory CD8 T cell subsets

The current most commonly used memory T cell subset denominations are central memory (Tcm), effector memory (Tem), peripheral memory (Tpm) and tissue‐resident memory (Trm), with the addition of effector memory RA + memory T cells (Temra) in humans.^19^ Of note, the phenotypic marker‐based classification of these subsets differs between murine and human T cells. For instance, circulating T cells in humans are predominantly classified using the markers CD45RA, CD45RO, and CCR7, while the classification of murine circulating T cells relies on CD62L and CX3CR1 among others.^19^ Thus, careful considerations need to be made when making assessments on the development of CD8 T cell memory while referring to the named CD8 T cell memory subsets.

One of the first descriptions of memory subsets was reported by Hamann et al, who noticed differences in phenotypic properties (CD45RA and CD27) among primed subpopulation of CD8 T cells in healthy human blood and found that those subpopulations had distinct functional properties.^1^ Sallusto et al described a similar phenomenon also in humans, but used the chemokine receptor CCR7 for sub‐setting antigen‐experienced T cells.^2^ Since CCR7 is required for entry into non‐inflamed lymph nodes through high endothelial venules (HEV),^20^ antigen‐experienced CCR7^+^ T cells are thought to be primarily involved in surveying secondary lymphoid organs and are referred to as central memory T cells (Tcm).^2^ A similar subset was identified in mice, when it was observed that murine antigen‐experienced CD8 T cells can be sub‐divided into CD62L^+^ and CD62L^–^ cells.^21^ Since immune cells require both CCR7 and CD62L to migrate across HEV, and the murine CD62L^+^ memory subset exhibited similar functional properties as the human CCR7^+^ subset, Tcm today are most commonly defined as memory CD8 T cells expressing CD62L and/or CCR7.

In both humans and mice, the populations of memory T cells lacking CD62L and/or CCR7 had more enhanced effector and cytotoxic functions in short‐term assays and are therefore most commonly referred to as effector memory (Tem).^2^, ^21^ Over the years, it has however become clear that neither the CD62L^–^CCR7^–^ population, nor the CD62L^+^CCR7^+^ population is functionally homogeneous, and thus that additional heterogeneity exists among memory T cells.^22^ Examples of additional molecules that have been shown to be differentially expressed among memory CD8 T cells are CD43, KLRG1 and CX3CR1.^23^, ^24^, ^25^, ^26^ CX3CR1 is noteworthy in that it is not a bimodal marker like the others, but distinguishes three subsets based on a gradient of expression levels.^24^ CX3CR1^–^ memory T cells largely overlapped with the lymph‐node homing CD62L^+^ Tcm population both functionally and phenotypically, while all CX3CR1^hi^ memory T cells were CD62L^–^. Interestingly, the CX3CR1^int^ population consisted of both CD62L^–^ and CD62L^+^ cells, but was shown to have unique functional, homeostatic (turnover rate), and migratory properties; The CX3CR1^int^ population is referred to as peripheral memory (Tpm) cells given they are the predominant population circulating between blood and peripheral tissues. The CD62L^–^CX3CR1^hi^ population, which corresponds to the memory population with the highest immediate cytotoxic ability, is restricted to blood and splenic red pulp. In light of this, CX3CR1 is an additional useful marker as it delineates both effector and memory CD8 T cell subsets more finely and is able to stratify them based on the degree of their differentiation, homeostatic properties, and migratory patterns.

Exploring the heterogeneity in the subsets lacking CCR7 and/or CD62L also led to the discovery of non‐circulating CD8 memory T cells that resided long‐term in non‐lymphoid tissues.^27^, ^28^ These were aptly named tissue‐resident memory cells (Trm). Methods of identifying Trm use carefully titrated amounts of intravenously injected antibodies to deplete circulating adoptively transferred T cells using antibodies directed against congenic markers (eg Thy1 or CD45 isoforms) on the adoptively transferred T cells^29^, ^30^ or rely on parabiosis experiments,^31^ in which mice with distinct congenic markers are surgically joined to permit sharing of blood between the two animals. Therefore, circulating T cells equilibrate between the two animals, while the tissue resident T cells do not re‐circulate and thus do not cross over to the joined partner.^32^, ^33^ Since such assays are not always possible, a commonly used surrogate to identify Trm in both humans and mice relies on the phenotypic markers CD69, CD103 and CD49a, which are molecules that play a role in cell retention in tissues^34^ and extravasation into tissues.^28^, ^35^ However, how useful these markers are to identify Trm depends on the tissues in which they reside.^36^, ^37^, ^38^ Further studies into Trm identified a core transcriptional profile common to this subset across different tissues, but distinct from circulating T cells.^35^, ^39^, ^40^ However, to establish functional tissue residency, parabiosis experiments or those using antibodies or other agents to deplete circulating cells are currently the most accurate methods to identify the Trm subset.

The vast heterogeneity found in CD8 T cell responses, and the parallel usage of functional, migratory and phenotypic subset delineations highlights the need for a more unified definition of the various T cell subsets, as has been discussed at the 2020 Keystone Symposium on T cell memory. This will help us to get a better appreciation of how the different CD8 T cell subsets work in concert to provide long‐lasting protection.

### 2.2 Origins of memory CD8 T cell subsets and their relation to effector subsets

With the identification of different memory subsets, questions on the origins of these subsets and the mechanisms by which they develop started to arise. At what point during a response do naive T cells acquire distinct functional and migratory properties and populate the various memory pools? Efforts to address these questions led to investigating which subsets from the effector phase of an infection influence the generation of memory T cells. Initial studies found that T cells in the effector phase required IL‐7 to survive into the memory phase^41^ and that these effector cells expressed varying levels of the IL‐7 receptor alpha chain (IL‐7R or CD127), which could influence whether they will enter the memory pool or not.^42^ In addition, early effector cells expressing IL‐7R and KLRG1, the latter controlled by IL‐12 and T‐bet levels, were found to be less likely to survive into the memory phase as compared to their IL‐7R^+^, KLRG1^‐^ counterparts.^25^ However, further studies demonstrated that factors other than IL‐7 responsiveness are also involved in regulating effector cell survival into the memory pool and that additional phenotypic markers may be used to delineate those effector cells with a higher or lower likelihood to form memory.^24^, ^43^, ^44^, ^45^ As such, it is not yet well‐defined why certain cells have the ability to survive long‐term while others do not. Furthermore, it has become clear that the tissue microenvironment plays a role in the development and maintenance of Trm cells,^37^ and whether it additionally contributes to the formation of circulating memory T cells is also something to consider.

An aspect that further complicates our understanding of developmental relationships of memory T cell subsets is the possibility of plasticity between the different subsets. For instance, some CD62L^–^ memory CD8 T cells were found to eventually re‐express CD62L and produce IL‐2 in steady state.^21^, ^24^ Similarly, circulating CX3CR1^int^ memory cells could convert to CX3CR1^–^ cells under steady state. Additionally, a recent study demonstrated how KLRG1^+^ IL‐7Rα^+^ effector cells can downregulate KLRG1 and differentiate into all memory subsets in response to secondary *Listeria monocytogenes* infection.^46^ Furthermore, after re‐exposure to antigen, CD4 and CD8 Trm cells may leave their tissues of prior residence and join the circulating pool.^47^, ^48^, ^49^ Whether these changes in functional and migratory characteristics can be classified as subset plasticity or (de‐)differentiation is a subject for debate. The context in which these changes occur is important to consider and will provide insight into the true nature of these subsets.

The complex nature of effector to memory subset transition, and the possibility of plasticity and de‐differentiation all point to the importance of studying individual T cells to monitor the development of T cell subsets within a population. In this regard, utilizing single‐cell technologies to fate map and monitor development of T cell subsets will help address how individual naïve T cells contribute to the diverse effector and memory pools. Moreover, the population dynamics of these subsets can be more closely monitored and a holistic and more detailed picture on the development of these various effector and memory subsets can be formed.

## 3 CURRENT TECHNOLOGIES TO FATE MAP AND TRACE T CELL SUBSET DEVELOPMENT WITHIN POPULATIONS

**FIGURE 2:**
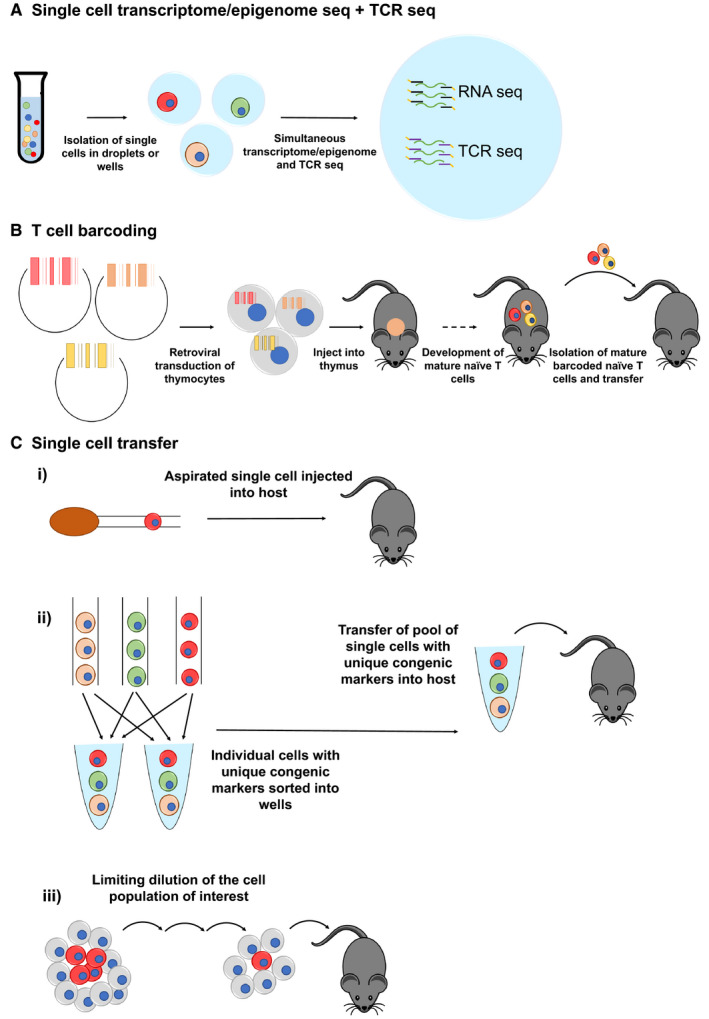
Single‐cell techniques for fate mapping and lineage tracing of T cells Illustrations of the current single‐cell techniques available to perform lineage tracing and fate mapping experiments of individual naïve T cells. A) Single‐cell transcriptomic or epigenomic profiling including TCR sequencing of individual cells. Single cells are isolated from a suspension by sorting using flow cytometry or microfluidic techniques into droplets containing uniquely tagged primers amplifying the cell’s transcriptome or epigenome. The droplets could also contain TCRα and TCRβ chain specific primers with the same unique tags (reviewed in Ref 85). B) T cell barcoding relies on using a plasmid library containing a pool of unique DNA sequences. This library is then retrovirally transduced into congenic thymocytes at a rate ensuring one plasmid per cell. These thymocytes are then transferred directly into thymi of mice to generate mature naïve barcoded T cells within that mouse. The barcoded naïve T cells are then isolated and subsequently transferred into an adequate host.^8^ C) Single cell transfer into mice has been conducted using three different techniques. i) A single naïve T cell with a unique congenic marker is aspirated into a needle under microscopic control and its presence is also verified by microscopy. With the same needle, the cell is transferred into mice intraperitoneally.^9^ ii) Through successive rounds of single‐cell sorts using flow cytometry, with each round sorting naïve T cells with a different congenic marker into the same collection well, a pool is generated containing one naïve T cell per unique congenic marker. This pool is subsequently transferred into one mouse intraperitoneally.^14^ iii) Transfer of an individual T cell using limiting dilution. The frequency of T cells with a certain antigen specificity is determined in a naïve population. The population is diluted accordingly to ensure the frequency of the naïve T cell of interest occurs so that only one T cell of interest is transferred^15^

Monitoring and assessing the behaviour of individual T cells within a population throughout the course of an infection requires the ability to identify individual T cells and link the progeny of individual cells back to their ancestors. A number of technologies have been developed which enable one to achieve this (summarized in Figure 2). Application of these technologies has led to novel and insightful discoveries on the contribution of individual naïve T cells to the overall immune response and the development of effector and memory CD8 T cell subsets. In the following section, a selection of fate mapping and lineage tracing technologies allowing assessment of cells within a T cell population at the single‐cell level over the course of an infection will be described and their contribution to the field highlighted.

### 3.1 Single‐cell sequencing including T Cell receptor (TCR) sequencing

Single‐cell transcriptome and epigenome sequencing including TCR sequencing (Figure 2A) capture the transcriptomic or epigenomic landscape of an individual cell at a certain point in time, while simultaneously providing the ability to identify which T cells are progeny of the same naïve T cell based on the TCR sequence. It is necessary to combine the two sequencing methods in order to fate map and lineage trace cells; cells with a specific TCR will clonally expand when encountering their cognate antigen, thus the progeny of a given T cell with a specific TCR sequence can be monitored over time. It is important to realize though that in order to unambiguously determine whether expanded T cells derive from a common individual naïve T cell, both TCRα and β sequences need to be evaluated; thymocytes undergo multiple rounds of division after completing rearrangement of the TCRβ chain before recombining the TCRα chain and forming a complete TCR. Thus, TCRβ sequences are not necessarily unique to individual naïve T cells (discussed in Ref. (50)). This combination of sequencing techniques has the capability of capturing the transcriptomic or epigenomic heterogeneity within a population at single‐cell resolution and also traces the cells’ origins to a precursor T cell with a given TCR sequence.

Single‐cell sequencing^51^ requires isolation of single cells—usually by flow cytometric sorting or single‐cell containing droplet formation—and the subsequent amplification of each cells’ transcriptome or epigenome, with the cells’ identity encoded by a unique tag per cell. These single‐cell libraries are then pooled, amplified and sequenced using next‐generation sequencing technologies.

#### 3.1.1 Single‐cell transcriptome sequencing combined with TCR sequencing

The combination of TCR sequence information and transcriptional state of individual T cells has had significant impact on highlighting heterogeneity in antigen‐specific populations, while simultaneously establishing kinship among individual cells and identifying a T cell's reactivity towards a given antigen. For example, tumour‐specific and non–tumour‐specific CD8 T cells from the same human melanoma patient displayed highly diverging states of exhaustion or dysfunction, which correlated with clonal population size.^52^ Clone size was also associated with the transcriptional state of murine CD8 T cells responding to vaccination, whereby clones could be grouped based on the gene modules they preferentially expressed.^53^ Transcriptional heterogeneity was furthermore demonstrated within and between CD8 T cell clones responding to Yellow Fever Virus vaccination in human volunteers (https://www.biorxiv.org/content/10.1101/832899v2), and within expanded antigen‐specific CD4 T cell clones in patients with a peanut allergy^53^ and colorectal carcinoma.^54^ The latter study found that two clones that made up 10% of the total intra‐tumoral CD4 T cell population specific to a peptide‐MHC ligand were comprised of progeny expressing combinations of IL‐17, RORC (which encodes RoRγT) and FOXP3.^54^ The phenomenon that few T cell clones dominate antigen‐specific responses and that individual clones consist of ‘T cell family members’ with highly diverse properties had previously been reported for murine CD4 and CD8 T cells responding to infections using other technologies^8^, ^9^, ^12^, ^13^, ^14^, ^15^, ^55^ (see barcoding, single cell transfer), and these single‐cell RNA‐sequencing‐based studies extend this concept to human tumour‐, allergen‐ and vaccine antigen‐reactive T cells.

The combination of single‐cell transcriptome sequencing with TCR sequencing also has the potential to investigate clonal diversity within memory T cell subsets to determine clonal origins and turnover within the subsets. A study comparing TCRβ sequences within populations of CD45RA^+^/CCR7^+^ (‘naïve’), CD45RA^–^/CCR7^+^ (‘Tcm’), CD45RA^–^/CCR7^–^ (‘Tem’) and CD45RA^+^/CCR7^–^ (‘Temra’) CD4 and CD8 T cells isolated from different human tissues revealed interesting differences between CD4 and CD8 T cells with regard to clonal diversity^56^; For example, the CD8 CD45RA^–^/CCR7^–^ subset displayed reduced clonal diversity compared to the CD4 CD45RA^–^/CCR7^–^ subset, and the CD8 CD45A^–^/CCR7^–^ clones were shared between different tissue sites, while CD4 CD45RA^–^/CCR7^–^ clones were tissue restricted. Reduced clonal diversity in CD8 T cells as compared to CD4 was also observed among Trm (CD69^+^) populations within tissues.^40^ Clonal overlap between different tissues for both CD4 and CD8 Trm was low, indicating that Trm may be more clonally segregated within the tissues in which they reside.^40^ Although these latter studies determined TCR diversity within T cell populations, they demonstrate the potential power of combining single‐cell RNA‐sequencing with TCR sequencing, and the wealth of information on the heterogeneity and clonality of different CD8 T cell subsets this combined technologies can provide. While we are not aware of any published work in this regard, such studies are on their way; work deposited on a preprint server reports that different human CD8 T cell clones responding to Yellow Fever Virus vaccination are skewed towards preferential production of different memory subsets (https://www.biorxiv.org/content/10.1101/832899v2). We also expect the rapid emergence of studies combining single‐cell transcriptome sequencing, TCR sequencing and sequencing of oligonucleotide‐tagged antibodies bound to the same individual cells to obtain paired protein‐expression data^57^, ^58^ to study clonal aspects of T cell responses, since this combination of technologies is now commercially available. Likewise, spatial transcriptomics^59^ has become commercially available—a technology that provides position‐specific transcriptomic data from tissue sections through the use of position‐unique primers and thereby links gene expression to defined regions within a tissue. Given that T cell subsets have distinct migratory properties, and that tissue sections better reflect the actual abundance and representation of T cell subsets than T cell suspensions obtained from disrupted tissues,^38^, ^60^ spatial transcriptomics may add valuable insight into the diversity of local T cell responses. The relative scarcity of antigen‐specific T cells however limits the amount of antigen‐specific T cells that can be examined within a single section. While the spatial resolution is not yet at the level of single cells, this will likely develop to reach higher resolution in the future.

#### 3.1.2 Single‐cell epigenome sequencing combined with TCR sequencing

The recent combination of TCR sequencing with single‐cell assay for transposase‐accessible chromatin (ATAC)‐sequencing enabled linking epigenetic states to clonal responses. ATAC‐sequencing identifies ‘open’ chromatin regions that are accessible for transcription. Epigenomic profiles provide additional insight into a cell's differentiation state and can reflect on the plasticity of a cell's current state and how easily it can transition to a different one. Transcriptional and epigenomic profiles of T cell subsets have been shown to not overlap in two‐dimensional representations of the high‐dimensional sequencing data.^48^ Furthermore, comparisons between phenotypic, transcriptomic and epigenomic profiles indicated that Trm share phenotypic signatures with effector cells, but have more of a resting state epigenetic signature. Satpathy et al employed this technology as a proof of concept to examine the epigenomic states in relation to the TCR of individual T cells isolated from patients with cutaneous T cell lymphoma.^61^ They found the majority of the CD4 T cells isolated expressed a single TCRαβ sequence, indicating that these cells may have arisen from a single leukaemic T cell clone. Epigenomic sequencing allowed for the identification of different profiles of accessible transcription factor binding motifs which enabled the characterization of cell types. Individual cells in the expanded T cell population displayed a profile associated with memory cells in addition to the accessibility of Th2 specific transcription factor GATA3—an effector subtype of CD4 T cells, while the non‐expanded T cell populations displayed a profile associated with naïve cells. In this study, the expanded T cell population was comprised of cells with a more or less homogenous profile. This study demonstrates that a combination of epigenetic profiling and TCR‐seq in a single cell can in fact be done. Although this has not been used yet to interrogate T cell subset differentiation, it has the potential to do so and can identify degrees of differentiation and potential function of T cell subsets within clonal populations.

The strength of the above‐described technologies lies in the high dimensional information on a cells’ transcriptional and epigenetic state, while linking the assessed state to the clonal origin of the cell. In this way, one can probe immune cell populations and highlight heterogeneity that could not have been discovered relying on a few phenotypic markers alone, as assessed by flow cytometry.^40^, ^61^, ^62^, ^63^ The immense amount of data generated by these sequencing approaches is on one hand its strength, but on the other hand it also calls for sophisticated and dedicated analysis to ensure accurate interpretation of the data. Furthermore, the cells are destroyed upon analysis and therefore cannot be used in subsequent analyses (eg functional assays or assays assessing the cells’ developmental potential), and so the data obtained correspond to a snapshot at a given time. It is also important to note that not necessarily all T cell expressing the same TCR sequence reflect the progeny from a single naïve T cell, as naïve T cells with the same TCR sequence occur at varying frequencies within the naïve population.^64^ This needs to be considered when assessing T cell fate in relation to the TCR, as studies have shown that even within a TCR transgenic population (T cells express the same TCR), individual naïve T cells behave differently upon activation.^8^, ^9^, ^13^, ^14^, ^17^, ^55^

### 3.2 T cell barcoding

T cell barcoding (Figure 2B), as developed by the lab of Ton Schumacher, is a technology that allows fate mapping of individual naïve T cells even among T cells expressing the same TCR sequence, which allows assessment of intra and inter‐clonal heterogeneity with respect to the TCR that is irrespective of differences in the T cells’ affinity for antigen.^8^, ^13^, ^16^, ^65^, ^66^ The technology uses a plasmid library in which each plasmid contains a unique DNA sequence, a ‘barcode’. To avoid activation of naïve T cells for barcode‐labelling by retroviral transduction, this plasmid library is retrovirally transduced into a population of naturally cycling thymocytes under conditions ensuring each cell gets one barcode. The barcode‐labelled thymocytes are then injected into the thymus of unmanipulated host mice to allow the development of mature naïve T cells from the transduced thymocytes. A selection of barcode‐labelled T cells is then transferred into a suitable host to track their behaviour in response to antigen challenge, while taking care that each transferred T cell harbours a unique barcode. Barcode sequences present in isolated T cell populations—possibly sorted by flow cytometry into subsets—may be determined using microarray or sequencing‐based readouts, with the sequencing readout additionally enabling relative quantification of barcode abundance, which relates to the amount of T cells harbouring this barcode. Using this technology, it was demonstrated that a single naïve CD8 T cell can give rise to both effector and memory T cell progeny regardless of TCR affinity, priming site and also under conditions of systemic and local infections or vaccination.^8^, ^13^, ^16^ Furthermore, this technology was able to reveal that while the pattern and even magnitude of T cell expansion and contraction are highly reproducible when a T cell *population* was tracked, *individual* naïve T cells harbouring the same T cell receptor actually contributed to the overall T cell response in highly unequal proportions.^13^, ^16^ Moreover, these individual naive T cells produced progeny with distinct phenotypic properties, such as differing KLRG1, CD27 and CD62L expression levels, where the fraction of the progeny with a given phenotype was somewhat proportional to the size of the family generated from each naive T cell.^13^ Individual naïve T cells also produced progeny with the capacity to migrate to various tissue sites, but while all clones were equally represented in different locations during the effector phase, about half of individual clones exhibited a bias towards preferential production of either Trm or circulatory memory T cells.^16^ The reproducibility of a T cell response can therefore be attributed to individual T cell responses being averaged over a population.

The advantage of this technology is the ability to assess inter‐clonal differences that exist irrespective of a T cells’ affinity for antigen and to monitor the contribution of currently up to hundreds of individual naïve T cells to different effector and memory T cell subsets within the same animal. However, a setback is that—like with other sequence‐based readouts—cells are destroyed in order to identify the barcode. Therefore, T cells whose barcode is known cannot be further assessed in, for example, functional assays, nor analysed more than once.

### 3.3 Single cell transfers

Another method of tracking individual T cells and their progeny is by transferring a single T cell into a suitable host, with the transferred cell and the host being distinguishable by expression of distinct congenic markers. This method has been pioneered by the lab of Dirk Busch and relies on isolation of single naïve T cells under microscopic control, and their subsequent transfer to a host. Using this technology, it was demonstrated that a single naïve T cell is capable of producing highly diverse effector and memory T cell subsets in response to bacterial infection.^9^ Due to the extremely low throughput of this method (1 single cell is tracked per mouse), it was later developed further to transfer up to 7 single naive T cells each with unique congenic marker combinations.^14^ Similar to what was shown using T cell barcoding, this study demonstrated that individual naïve T cells have differing expansion patterns, where the progeny of each naïve T cell contributes to the overall T cell population with unequal frequencies. Moreover, the degree of expansion from each T cell corresponded to different phenotypic properties, where individual naïve T cells that produced a large number of progeny produced predominantly CD62L^–^ CD27^–^ T cells. Single‐cell transfers have also allowed for the investigation into the plasticity of memory T cell subsets. Single‐cell transfers of CD44^hi^, CD62L^+^ CD8 (‘Tcm’) showed that individual cells are capable of producing progeny with diverse migratory characteristics and effector behaviour, demonstrated by the presence of populations with different CD27 and CD62L expression. Moreover, single CD44^hi^, CD62L^+^ CD8 T cells maintained this plasticity throughout serial single‐cell transfers and were capable of restoring immunocompetence in Rag2 knockout mice^55^

An alternative method of transferring a single T cell into a host is the limiting dilution method. This technique relies on determining the frequency of the antigen‐specific T cell population of interest within a naïve population, and adjusting the total number of cells transferred to ensure only one antigen‐specific T cell is included in the transferred population. This method was used to examine the effects of the strength and duration of TCR:peptide‐MHCII interactions on CD4 T cell differentiation,^15^ and to examine the role the tissue microenvironment plays in shaping CD8 T cell effector responses,^12^ and equally led to the conclusion that considerable heterogeneity exists both between clonal progenies and among the progeny of a single naïve T cell.^12^, ^15^, ^67^

The advantage of using single‐cell transfer techniques is that they provide irrefutable evidence as to which cells give rise to the observed T cell progenies after an infection—a feature that is shared by the barcoding technology. An advantage over sequencing‐based readouts like with TCR sequencing or T cell barcoding technology is that the transferred cell and its progeny do not need to be destroyed upon analysis; the unique congenic markers can be identified using flow cytometry along with other phenotypic and functional markers, providing more information on the phenotypic and functional state of the cell at that time point. Furthermore, the transferred cell and its progeny can be isolated, re‐transferred, and re‐analysed, which would be useful for examining plasticity of memory subsets for example.^55^ However, the single cell transfer methods used by Stemberger et al and Buchholz et al are technically challenging, and with the limiting dilution method there is no guarantee that a single cell has in fact been transferred. Furthermore, both single‐cell transfer methods, and in particular the limiting‐dilution‐based one requires a very large number of mice to ensure that enough cells are recovered to make valid statistical and scientific claims.

### 3.4 Single‐cell fate mapping in vitro

In vitro methods for tracing individual cells have equally highlighted factors contributing to cell fate within a population. Such methods rely on seeding either individual T cells or combinations of several uniquely labelled T cells into (micro)wells, and subsequently examining their progeny using live‐cell imaging, flow cytometry, or both. Alternatively, individual T cells have been expanded in microfluidic devices under continuous live imaging, followed by controlled release of the individual cells for subsequent single‐cell RNA sequencing.^68^ The latter method allowed coupling of an individual cell's transcriptional state to its division history and family relationship to other cells.

Similar to what has been shown for in vivo T cell activation, in vitro primed single naïve CD4 and CD8 T cells also gave rise to phenotypically and functionally diverse progeny; clone‐to‐clone variation was observed with regards to average progeny phenotype, function, transcriptional state and clonal expansion.^18^, ^67^, ^68^, ^69^ Inter‐clonal diversity was remarkably more pronounced than intra‐clonal diversity, suggesting a degree of heritability of cell fate.^67^, ^68^, ^69^, ^70^ Specifically, in vitro single‐cell fate mapping studies observed heritability of CD25, CD62L, CD8, Granzyme B, Blimp‐1,^68^, ^69^ cell cycle speed^69^, ^71^ and time until cessation of proliferation.^70^ It is at this point unclear though whether the heritability and fate symmetry observed in these in vitro systems reflects what happens during in vivo initiated responses, since depending on the in vitro stimulation conditions, T cells may undergo asymmetric or symmetric divisions,^72^ and the priming conditions used in the described clonal fate mapping studies were promoting primarily symmetric divisions.

The advantage of using an in vitro system is the ability to address extremely detailed questions on the influence of specific processes on cell fate and cell differentiation. Moreover, questions on how cell–intrinsic processes or external soluble factors can influence cell fate can also be more easily controlled for in vitro rather than in vivo. One obvious disadvantage is that in vitro systems are essentially artificial, and whether the processes discovered in vitro truly reflect those occurring under physiological conditions will always be an issue to address.

The current single‐cell T cell fate mapping technologies have demonstrated their potential in revealing inter‐clonal heterogeneity and variable clonal contributions to effector and memory T cell subset generation, as well as probing plasticity and differentiation states of these different subsets. These technologies have provided insight into the development of T cell responses in a manner that cannot be achieved using other technologies, thus furthering our understanding in the diversity and complexity of the T cell response.

## 4 FUTURE TECHNOLOGIES

The current single‐cell technologies have improved our understanding of the development of effector and memory CD8 T cell populations with reference to their heterogeneity and plasticity. Questions still remain on what factors influence individual naïve T cells to populate effector and/or memory T cell pools to varying degrees. How do individual naïve T cells combine signalling cues—from the T cell receptor, co‐stimulatory factors and cytokines provided by antigen presenting cells and the local microenvironment—to generate the diverse effector and memory subsets observed? At least one requirement to answer such questions is the ability to record past signalling events. The current single‐cell fate mapping and lineage tracing technologies discussed cannot answer such questions, and new approaches are required to address them.

One method of integrating signalling events to address their role in the development of effector and memory T cell subsets is to combine the above‐described technologies with transgenic reporter mice—which have so far only been used to track the fate of T cell *populations*.

Transgenic reporter mice have been generated to address questions on the behaviour and developmental requirements of certain T cell subsets on a population level, as well as to address changes of effector functions of T cell populations.^73^ This is achieved by coupling signalling events to the expression of a detectable marker, such as a fluorescent protein. In reporter systems where expression of the marker gene is controlled by the promoter of a gene of interest, for example a cytokine, marker expression is transient and roughly correlates with the duration of expression of the gene of interest. In other reporter systems, marker expression is permanent and heritable and therefore allow fate mapping. Such fate mapping reporters can be designed by, for example using the Cre‐loxP recombination system^74^; Cre recombinase expression is driven by the promotor of a protein or cytokine of interest, or by a drug inducible promoter. Cre expression in turn causes excision or inversion of a loxP flanked region, resulting in the permanent expression of a marker gene. Thus, current and past expression of a certain signalling event is reported permanently.

An example of such a transgenic fate mapping reporter is a mouse in which current and past IL‐17A production is reported by permanent expression of YFP. Thus, cells that produce or have produced IL‐17 would permanently be marked by the expression of YFP. This mouse has for example been used to investigate the plasticity of CD4 Th17 cells (defined as producing IL–17) to switch to exhibiting other effector functions.^75^, ^76^ Additionally, studies using granzyme B and IFNγ fate mapping reporter mice demonstrated that memory T cells can be generated from cells that have previously expressed these transcripts during an infection^77^, ^78^, ^79^

Transgenic fate mapping reporter mice have also been used to assess the contribution of CD8 T cell generated from foetal or adult haematopoietic stem cells to effector and memory populations, and it was found that CD8 T cells derived from foetal haematopoietic stem cells are ‘pre–programmed’ to mount an effector response and display an effector like chromatin landscape.^80^

Other methods of recording signalling events would be to incorporate methods developed by synthetic biologists who have made extensive progress in the development of synthetic‐based memory.^81^, ^82^, ^83^ Systems coupling DNA modifying approaches with signalling events may be adopted to address questions on the signalling events influencing the development of CD8 T cell subsets. For example, a novel system based on the CRISPR‐Cas9 system named CAMERA (CRISPR‐mediated analog multi‐event recording apparatus) was developed to record multiple cell states such as exposure to antibiotics, nutrients and light and translate this into changes in the DNA of bacterial and mammalian cell systems. In addition, the duration and strength of the signals received were reflected in the degree of DNA editing.^84^

The advantage of the transgenic reporter and synthetic biology systems is that cells are permanently marked by prior signalling events. These events can also be recorded in a non‐invasive manner, thus allowing populations to be tracked and analysed multiple times. This permanent mark is also heritable, which allows for monitoring progeny based on a past signalling event. This however can also be a disadvantage, since a cell that has already inherited a recombination‐induced permanent marker cannot record the same signalling event again, unless the fate mapping reporter system is combined with the transient reporter system. Moreover, synthetic biology techniques still need to be optimized for use in whole organisms. Combining these systems with the single‐cell technologies discussed in the previous section will provide an additional dimension in understanding how heterogeneity within T cell responses is generated, and to integrate the influence of past signalling events of individual naïve T cells with the development of effector and memory T cell subsets.

## 5 FINAL CONCLUSIONS

This review discussed the current technologies available for lineage tracing and fate mapping of single naïve T cells in the context of the development of heterogeneity within T cell responses. We envision that a combination of the current technologies will further advance our understanding of how individual naïve T cells and their progeny contribute to the highly diverse effector and memory CD8 T cell subset pools. New technologies are needed to address the mechanisms by which these individual naïve CD8 T cells and their progeny end up in said pools. Such new technologies will likely help address fundamental questions in CD8 T cell memory subset generation, furthering our understanding on the complex biology of the adaptive immune system.

## CONFLICT OF INTEREST

The authors declare no conflicts of interest.
